# Carers' experiences and perspectives of the use of anticholinergic medications in people living with dementia: Analysis of an online discussion forum

**DOI:** 10.1111/hex.13972

**Published:** 2024-01-15

**Authors:** Bara'a Shawaqfeh, Carmel M. Hughes, Bernadette McGuinness, Heather E. Barry

**Affiliations:** ^1^ School of Pharmacy Queen's University Belfast Belfast Northern Ireland UK; ^2^ Faculty of Pharmacy AL‐Zaytoonah University of Jordan Amman Jordan; ^3^ Centre for Public Health Queen's University Belfast Belfast Northern Ireland UK

**Keywords:** anticholinergic drugs, carers, dementia, online discussion forum

## Abstract

**Introduction:**

There is concern about the use of anticholinergic medications in people living with dementia (PLWD). Such medicines may increase cognitive decline and may be associated with higher mortality in PLWD who take these medicines. The aim of this study was to analyse data from an online dementia discussion forum to explore the experiences and perspectives of PLWD and carers about the use of anticholinergic medicines in this population.

**Methods:**

Following receipt of ethical approval, archived discussions (posts) from Dementia Talking Point, a fully public online forum for anyone affected by dementia, created and maintained by the Alzheimer's Society, were searched from the date of inception to January 2022 using a range of search terms including commonly used anticholinergic medicines. Posts, including any of the search terms, were assessed for relevance and analysed using inductive thematic analysis.

**Results:**

Five hundred and fifty unique posts were analysed, all of which had been provided by carers, with no posts attributed to PLWD. The themes that encompassed carers' experiences were (1) motivators of prescribing, (2) perspectives on the process of prescribing and (3) the outcomes of prescribing. The dominant motivator of prescribing was the management of noncognitive symptoms, pre‐ and postdiagnosis of dementia. Carers' perspectives on the process of prescribing were informed by an assessment of the risk‐benefit of starting a medication and shared decision‐making between the carer and healthcare professional to a greater or lesser degree. The outcomes of prescribing were observing the effects of the medicines, which in turn influenced whether prescribing was reviewed and continued unchanged, continued but amended, reinitiated if the medicine had been previously stopped or discontinued (the process of deprescribing).

**Conclusion:**

This study has provided unique insights into carers' experiences and perspectives about the use of anticholinergic medications in PLWD, highlighting how commonly these medications are prescribed for PLWD and carers' concerns about their use. There is a clear need for carers and PLWD to receive information about these medicines and healthcare professionals to consider how to optimise the use of these medicines to avoid adverse effects.

**Patient or Public Contribution:**

This work was informed by findings from previous research studies focusing on optimising medicine use for people with dementia in primary care, in which interviews were conducted with PLWD, their carers and primary healthcare professionals. Although not strictly patient and public involvement, we utilised the feedback provided by key stakeholders to inform the research questions and aim/objectives of this study.

## 1 INTRODUCTION

The use of medicines is one of the mainstays of management in the care of people living with dementia (PLWD).^1^ Numerous studies have been conducted to explore the perspectives of healthcare professionals (HCPs, e.g. pharmacists, general practitioners [GPs], nurses) about prescribing medicines for PLWD and the challenges this population faces when managing their medicines.^2^, ^3^, ^4^ However, only a small number of studies have set out to explore the perspectives of PLWD and their carers about medication use and prescribing and how they can be supported in using medicines. A recent systematic review was conducted to identify the impact of interventions at hospital discharge to guide carers in medication management for PLWD.^5^ The review identified only five studies and emphasised the need for well‐designed interventions to be developed to aid and guide carers with medication management for PLWD.^5^ Cross et al.,^6^ in a qualitative study (involving carers, PLWD, GPs, nurses and pharmacists), reported that participants agreed that carers had an important role to play in medication management for PLWD, acting as advocates, facilitating communication between HCPs and PLWD, helping with decision‐making and providing increasing assistance with medication administration as dementia progressed.^6^ All participants agreed on the importance of involving PLWD in medication management and decision‐making around medicines and that the role of carers was fundamental in medication management for PLWD.^6^

One category of medicines frequently prescribed in PLWD and which has given rise to concern is those with anticholinergic activity. Indeed, several hundred medicines may exhibit anticholinergic effects, which include drowsiness, blurred vision, dry mouth, confusion and hallucinations.^7^ However, a number of anticholinergic medicines are used to manage conditions such as urinary incontinence and prevention of blood clotting.^7^ Other anticholinergics are often prescribed to manage the noncognitive symptoms of dementia, such as aggression, agitation, wandering and mood swings. In the case of the latter symptoms, benzodiazepines (e.g., diazepam), antipsychotics (e.g., risperidone) and antidepressants (e.g., amitriptyline)^7^ may be used. However, there is growing evidence that their use may be associated with an increased risk of incident dementia.^8^, ^9^ A recently published observational study highlighted that higher anticholinergic burden (ACB—the cumulative effect of using multiple medications with anticholinergic properties concomitantly) was associated with significantly higher mortality rates in PLWD in comparison to PLWD who had no ACB.^10^ A systematic review found no eligible studies that aimed to reduce ACB among PLWD in primary care.^11^ This was an unexpected finding as it is recognised that interventions are needed to reduce ACB and the use of medicines with anticholinergic activity in this population without affecting the management of other conditions for which these medicines are prescribed.^12^

There is very limited literature describing the experiences and perspectives of PLWD and their carers about the use of anticholinergic medications in PLWD; a greater appreciation of these perspectives may help researchers and clinicians to better understand PLWD and carers' concerns about the use of these medications and facilitate their more judicious use by clinicians and prescribers. Online discussion fora are increasingly being used by researchers as they represent a rich source of data pertaining to patient and carer experiences and can often provide additional perspectives that would not be accessible by more conventional qualitative methods.^13^ Such fora have been utilised in research studies, including dementia and other long‐term conditions such as Parkinson's disease and stroke^14^, ^15^, ^16^ and are increasingly accepted as a source of qualitative data.^17^, ^18^, ^19^ The study outlined in this paper aimed to address this gap in the evidence base by analysing data from an online dementia discussion forum to explore the experiences and perspectives of PLWD and their carers about the use of anticholinergic medicines in this population.

## 2 METHODS

### 2.1 Setting

This study involved analysing archived discussions on Dementia Talking Point, a fully public online community for anyone affected by dementia, created and maintained by the Alzheimer's Society in the United Kingdom (https://forum.alzheimers.org.uk/). It contains fora, areas where discussions take place on different topics; within these, members can create a ‘thread’ (a group of posts identified by a title containing an opening or original post that opens the dialogue of discussion).^14^ The threads make it easier for users to find posts on a particular topic, such as people who are at a similar stage of dementia or in a similar situation. The threads can contain any number of posts, including multiple posts from the same members.^14^ Only those who are registered as members of Talking Point can create new threads, edit posts and receive notifications of replies. However, threads, posts and archived discussions can be viewed by nonmember visitors to the forum.^14^ According to the Talking Point website, there are currently 81,713 members, 130,483 threads and 1,903,747 posts.^20^ Since all members are required to state their reason for joining when registering to use Talking Point, it was assumed that they all had some experience with, or connection to, a person living with dementia.

### 2.2 Data selection

The researcher (B. S.) searched the archived Talking Point threads and posts to extract data for analysis; he did not create posts or contact any members of the forum. Threads from the date of inception (2005) of the Talking Point forum to the search date (January 2022) were searched by using keywords (search terms) within the advanced search facility provided by the forum. The search terms were informed by the literature, particularly three observational studies conducted in the United Kingdom,^8^, ^10^, ^21^ all of which reported commonly used anticholinergic medications amongst people with dementia. The search terms were discussed and agreed upon by the research team. The following terms, and combinations thereof, were used: ‘anticholinergic’, ‘antimuscarinic’, ‘antipsychotic”, ‘urological anticholinergic’, ‘oxybutynin’, ‘tolterodine’, ‘solifenacin’, ‘antidepressant’, ‘amitriptyline’, ‘diazepam’, ‘risperidone’, ‘paroxetine’, ‘dosulepin/dothiepin’, “quetiapine’, ‘isosorbide preparations’, ‘warfarin’. Examples of anticholinergic medicines and their main clinical indications are shown in Table 1.

**Table 1:** Examples of anticholinergic medicines.^22^

Medicine	Indication
Amitriptyline	Depression, neuropathic pain
Diazepam	Anxiety, insomnia
Dosulepin (brand name Dothiepin®)	Antidepressant
Isosorbide mononitrate/dinitrate	Angina, heart failure
Lorazepam	Anxiety, insomnia
Oxybutynin	Urinary frequency, urgency, incontinence
Paroxetine	Depression
Quetiapine	Schizophrenia, mania and depression in bipolar disorder
Risperidone	Psychosis, mania and depression in bipolar disorder
Solifenacin	Urinary frequency, urgency, incontinence
Tolterodine	Urinary frequency, urgency, incontinence
Trazodone	Depression, anxiety
Warfarin	Prevention of clotting

### 2.3 Data extraction and screening

All posts, including any of the search terms, were copied verbatim and transferred to Microsoft Word. The anonymity of forum members was assured by assigning a unique identifying code (e.g., TP001, where TP indicated ‘Talking Point’ and the number indicated the order in which posts were stored). Duplicate posts were removed. Posts were assessed for relevance to the study objectives by two researchers working independently (B. S. and H. E. B.). Irrelevant posts were removed, and reasons for exclusion were recorded. A third researcher (C. M. H.) was consulted when consensus could not be reached about the inclusion of a post.

### 2.4 Data analysis

Inductive thematic analysis was conducted by hand, using methods described by Braun and Clarke.^23^ Posts were coded to meet the study aims by identifying members' experiences of using anticholinergic drugs, their perspectives about reducing the use of anticholinergic drugs and their understanding of the risks involved with the use of anticholinergic drugs in PLWD. Coding was undertaken by B. S. with independent coding of a subsample (20%) of posts undertaken by H. E. B. Posts were categorised as being one of the following: unique posts, similar posts (i.e., posts in which the same story was repeated but not entirely identical wording was used) or similar posts with additional codes (i.e., posts in which the same story was repeated, not entirely identical wording was used but also contained additional details which generated additional codes). Coding was discussed amongst the research team until agreement was reached on the coding frame. These codes were aggregated into broader themes and then discussed and agreed upon by the research team. Illustrative quotations were used to support interpretations. Quotes were edited when needed to improve readability; where text has been added or clarification provided, this has been placed within square parentheses [].

Ethical approval was granted for this study by the Faculty of Medicine, Health and Life Sciences Research Ethics Committee, QUB on 4 January 2022 (Reference MHLS 21_160), and permission to use the forum data was granted by the Talking Point Manager. To enhance the reporting of this study, the COnsolidated criteria for REporting Qualitative studies checklist was used (COREQ)^24^ (see File 1).

## 3 RESULTS

**Figure 1:**
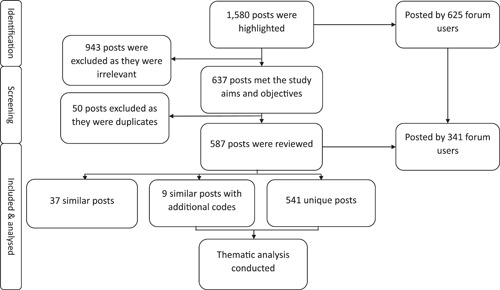
Flowchart summarising the process of data screening and selection.

Following the completion of the searches, a total of 1580 posts written by 625 forum users were extracted. Following the removal of duplicate posts, 587 posts were reviewed, and a total of 550 posts from 341 forum users were included for analysis. Among these, there were 541 unique posts and 46 similar posts. Of these similar posts, nine posts contained additional details, which generated additional codes. Therefore, these nine similar posts and the 541 unique posts were analysed (i.e., *n* = 550 in total). A flowchart of the process of data screening and selection is displayed in Figure 1.

An initial review of the posts indicated that they were provided exclusively by carers, and none could be identified as coming from PLWD. Therefore, the findings presented relate only to carers' perspectives.

**Figure 2:**
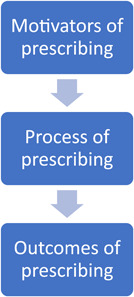
Forum users' experiences of the use of anticholinergic medications in PLWD.

Thematic analysis of the carers' posts revealed their experiences of the use of anticholinergic medications in PLWD. The themes that encompassed the experiences were as follows: (1) motivators of prescribing, (2) perspectives on the process of prescribing and (3) the outcomes of prescribing. The dominant motivator of prescribing was the management of noncognitive symptoms, pre‐ and postdiagnosis of dementia. The process of prescribing was informed by the assessment of the risk‐benefit of starting a medication and shared decision‐making between the carer and HCP to a greater or lesser degree. The outcomes of prescribing were observing the effects of the medicines, which in turn influenced whether prescribing was reviewed and continued unchanged, continued but amended or reinitiated if the medicine had been previously stopped or discontinued (the process of deprescribing). Figure 2 summarises the broad themes that encompassed the experiences of forum users, which are explained in further detail below.

### 3.1 Motivators of prescribing

Carers reported that the presentation of noncognitive symptoms such as aggression, agitation, wandering, changes to sleep patterns and mood disturbances in PLWD appeared to be the main motivator for prescribing anticholinergic medications:

My mother has advanced mixed dementia and has recently been prescribed risperidone for her aggression. (TP001)

My husband has advanced Alzheimer's & has lately been aggressive with his carers over his personal care, also has hallucinations, […] & is falling & unable to get himself up. Today, the Memory Clinic are thinking of prescribing risperidone. (TP003)

About a month ago, my husband was referred to a psychiatrist because of high anxiety, hallucinations and occasional bursts of violent behaviour with a view to putting him on risperidone. (TP005)

Indeed, some forum users reported that anticholinergic medications were used by PLWD before they received a dementia diagnosis in response to the presentation of noncognitive symptoms while observing that there had been a decline in cognition since starting the anticholinergic medications:

She is still taking the same tablets (high blood pressure and amitriptyline at night—very low dosage) that she was taking years ago before dementia started. (TP198)

Further decline has been progressive the past 6 months. She is on quetiapine 50 mg, which has been prescribed for a number of years due to insomnia. (TP304)

From the perspective of carers, the prescribing process after that was influenced by an assessment of the risk‐benefit of introducing medications, the need for trial and error and the approach taken to decision‐making between the PLWD, carer and prescriber.

### 3.2 Perspectives on the process of prescribing

Many information sources were reported to be used by forum users as they tried to understand the benefits and risks involved with using these medications if HCPs indicated that such medications might be required. Some were evidence‐based (e.g., clinical guidelines published by the National Institute for Health and Care Excellence^12^), but many were not, such as health websites and news stories that were not supported by robust evidence:

I have googled all the drugs, and lorazepam can cause them [PLWD] to be unsteady. (TP236)

Google all these drugs and find all the information about these drugs' side affects [sic]. (TP237)

There's a good page here which explains about trazodone and the possible side effects: [link provided]. (TP141)

In addition, many users asked fellow forum users for help to understand their views and experiences and the risks and benefits regarding the use of anticholinergic medications:

I don't know how long Mum has been taking these [oxybutynin] for. They are used for unstable bladders, and Mum has always had a problem with this score. Has anyone had any experience with these, please? (TP244)

Hi, I'm just wondering if anyone has had any experience with the drug [risperidone], good or bad? I have googled the side effects and was concerned when it says a risk of death in the elderly; it also carries more risks than some of the other antipsychotic drugs. (TP300)

It was also recognised that trial and error would be part of the prescribing process. Forum users reported that based on the effect of these medications, prescribers often had to adjust the frequency and dosing of anticholinergic medications, which forum users described as a process of trial and error:

The team have really investigated mum's condition, re‐confirm her diagnosis and with trial and error found the right medication for her. (TP090)

She was a lot better on risperidone; however, personally, I think it did push her quicker down the Alzheimer's road. But it is a balancing act, quality of life whilst they are here. (TP048)

It can be a case of ‘trial and error’ though to find the right balance between reducing the pain without over‐sedating, and mum had several changes of pain medication before the right dosage/medication was found. (TP016)

The knowledge of risks and benefits on the part of carers contributed to their understanding of decisions made by prescribers:

Yes, antipsychotics are not great for PLWD, but sometimes you have no other choice, and in your mum's case, the benefits might outweigh the risks. It's sometimes difficult to get the dosage right, but that doesn't mean meds shouldn't be given. Try to work with the doctors, not against them. (TP140)

I am glad they are trying her on these tablets & the possible side effects have been explained to me, but it's a case of weighing up benefits against risks. (TP262)

The extent of carer involvement in decision‐making was variable. As advocates, carers were keen to be a part of the decision‐making process since they knew the history of the PLWD and their medication history and could observe immediate and long‐term changes in a person's behaviour and symptoms. However, some carers described feeling excluded by HCPs from decision‐making about prescribing:

The private one [psychiatrist] insists on the drug that does not work even when I told her it does not work and makes mom restless. (TP046)

What I didn't know at the time, but now know, is that despite the nurse appearing to not have taken much notice of my concerns about him being on. When I spoke to her on Friday night, she'd with‐held it (amitriptyline) on Friday and over the weekend, and they'd cleared it with GP [general practitioner] to stop it first thing Monday. (TP618)

However, some carers involved in decision‐making reported that they felt stressed about making the wrong decisions in case this resulted in PLWD experiencing negative effects from initiated medications:

Last night, she had such an anxiety attack, and I think this [taking antidepressant medication] may help her. I hope so. I pray so. I am indeed fearful of doing the ‘wrong’ thing here. She never wanted anti‐depressive drugs, but she is crying frequently … (TP602)

### 3.3 Outcomes of prescribing

Following the initiation of prescribing of anticholinergic medicines, forum users described observing a variety of effects on PLWD:

After trying a few more medications, which all failed, we finally came to the conclusion that it was better not to use medication and to reduce the aggression by removing the things which caused the change in mood in the first place. (TP406)

My husband has been in care for nearly 2 years. Once he was put on risperidone, his health deteriorated, sometimes sharply, sometimes less so. (TP050)

Many carers described the negative effects of medications through the impact on their own levels of anxiety and quality of life, from having to deal with challenging behaviours and noncognitive symptoms, such as aggression:

Hi, OH [other half] been on risperidone almost 20 months following a harrowing time of delusions, accusing me of all kinds of unspeakable things, arguing day and night for weeks till I could get him to the doctors and onto the medication. (TP058)

My wife, when is [sic] was already clear that her dementia was well‐established, was prescribed amitriptyline because it was thought that she might be a bit depressed. Knowing that she has always been very sensitive to all drugs, I was advised to ‘start low’. I gave her 5 mg, the lowest prescription mentioned, last thing at night. She woke up raving. It was the most horrific experience I've had (so far). (TP200)

Some of the forum users reported that newly introduced anticholinergic medications were ineffective in managing noncognitive symptoms. Forum users described feeling frustrated when this happened:

My mum is now on her second type of antipsychotic, as the first had no effect. After a few good days initially, she is back to how it was before, angry, violent with me, everything is my faulylt [sic], wandering out now and then. (TP263)

Other forum users described the positive effects as ‘life‐changing’ for both carers and PLWD. Many forum users observed a major improvement in noncognitive symptoms after the introduction of an anticholinergic medication:

Mum became extremely violent towards us and cried a lot in the mid‐stages. Once the doctor prescribed an antipsychotic and an anti‐depressant, she became much calmer. Three years down the line, she is still on them, and as long as she doesn't refuse to take them, we mostly have a good life. (TP410)

My mum eventually went into care and was medicated, and the difference in her was incredible. The paranoia stopped, and she could enjoy life again. (TP429)

Forum users acknowledged that anticholinergic medications would affect individuals differently and that this was important to consider when assessing a person's response to medication:

My advice is simply to keep a close eye on her responses. Not everyone will have side effects, or they might not be severe. (TP008)

Everyone reacts differently to medication, so until they try something, it's impossible to say if a drug will be of benefit. Risperidone is supposed to be the antipsychotic of choice for Alzheimer's but is definitely not for everyone. (TP152)

The presentation of side effects led to a review of medication, which could result in a medication being continued, a change (increase or decrease) in dosing, a medication that had previously been stopped being reintroduced or deprescribing (withdrawal/stopping a medication):

Hi. My dad also has LBD [Lewy Body Dementia]. He had rivastigmine [indicated for mild‐moderate dementia] patches and quetiapine, both of which helped in the early stages. After bouts of aggression, these were both increased. (TP270)

Other forum users noted the disappearance of negative effects after reducing the dose of an anticholinergic medication:

That's what I did whenever Mum started getting sleepy. Each time, they reduced the dosage, and eventually, each drug was removed permanently. I strongly recommend you try that. A slightly reduced dosage may help keep her more alert while still keeping her reasonably calm. It worked for my mother. (TP008)

Some users observed the reappearance of noncognitive symptoms when the dose of an anticholinergic medication was reduced, and in some cases, the medication was reintroduced:

After a very difficult time, my husband was prescribed risperidone, which made our lives much easier. Dosage reduced to a minimal amount but noticed the aggressive moods returning. (TP045)

After initially settling really well on an antipsychotic drug, which she was taken off after only a few weeks, then put back on a few weeks later, she just can't seem to settle. (TP413)

Deprescribing (withdrawal/stopping medications as described by forum users) was initiated by different prescribers (such as secondary care consultants and GPs) and sometimes carers when they reported a negative effect from anticholinergic medications:

I stopped the medicine [risperidone], and in a few days, he was back to what is now normal for him … His doctor was surprised at how quickly he had the reaction and recovery. (TP083)

My MIL [mother‐in‐law] was prescribed a low dose of diazepam for anxiety by her GP about 18 months after diagnosis. But when she saw a specialist consultant, he said that the anxiety was a result of Alzheimer's, and the diazepam wouldn't help with that—he took her off [diazepam], and she was less confused and had fewer problems with incontinence. (TP166)

Some users also feared that following the deprescribing of anticholinergic medications, other comorbidities may become worse:

I wouldn't want to worsen her bladder control and maybe tip her into continence problems, but nor do I want her to take anything that might hasten the progress of her dementia. She's 90 now; if only I had a crystal ball to know how long she is going to be with us, it would make so many things easier. (TP244)

## 4 DISCUSSION

To our knowledge, this is the first study that has analysed data from an online discussion forum to understand carers' experiences without the use of anticholinergic drugs in PLWD. A total of 550 posts were reviewed and analysed thematically. This study showed that anticholinergic medications were being prescribed before a person receiving a dementia diagnosis. Forum users described assessing the risks and benefits associated with individual medications and the extent of their involvement in decision‐making. Finally, the outcomes of prescribing were reported on the basis of the effects of the medications and decisions being made whether to continue, change or discontinue medications.

In this study, the Talking Point forum proved to be a valuable source of data,^14^, ^25^ although it was evident that carers were exclusively represented in the posts extracted for this study. Rich accounts were collected from a large sample of ‘participants’ (341 unique forum users); the use of this method of data collection circumvented traditional approaches to sampling and recruitment normally followed in other qualitative study designs, which can be challenging in this population, and which would have been further complicated by the ongoing coronavirus disease 2019 pandemic at the time of this study. The data were collected in the absence of the researcher, which maintained the integrity of the data and anonymity of the participants, removed participant bias towards the research agenda and reduced the degree of intrusiveness.^13^, ^26^ However, due to the lack of interaction between the researcher and the participants (forum users), points that were unclear could not be clarified and the sociodemographic characteristics of study participants are unknown. In addition, forum users' posts were not always directed to the research question; therefore, the researcher spent a significant amount of time screening posts to ensure only relevant ones were included.^13^ This study found that anticholinergic drugs were commonly used by PLWD before they had received a dementia diagnosis, which is consistent with other studies reporting that older people had a high prevalence of anticholinergic drug use.^8^, ^27^, ^28^, ^29^ Forum users described that noncognitive symptoms were frequently observed in PLWD, which probably accounts for the prescribing of these medications and which reflects other published research.^30^, ^31^, ^32^ However, this contradicts clinical guidance and indicators of prescribing appropriateness, which recommend that anticholinergic medications should not be used in older people or those with dementia due to the increased risk of cognitive decline.^33^, ^34^

It was clear that Talking Point was considered a valuable source of information and support by forum users. However, there appeared to be no moderation of posts to evaluate the quality of the information provided by forum users or to remove irrelevant/incorrect information. Other studies have described the use of information sources, such as online fora, by patients and their carers when looking for answers to questions about their medical conditions.^35^, ^36^ However, this may suggest that carers are not being provided with sufficient information from HCPs regarding the availability of pharmacological and nonpharmacological management options and local services and support to which they could be signposted,^12^ leading them to look elsewhere for advice.

Prescribing often happens after the prescriber has considered the potential risks and benefits of these medications for the recipient. A key part of this assessment is considering the evidence base. However, based on the content of the forum discussions, it was not clear if HCPs adhered to the evidence. For example, the NICE guideline, which covers the management of dementia, recommends minimising the use of medicines, such as antipsychotics and antidepressants, associated with increased ACB in those with suspected and confirmed dementia and recommends that prescribers use alternative medications.^12^ Uptake and implementation of clinical guidelines is acknowledged to be challenging in clinical settings due to a lack of time, resources, and implementation support guidance, as well as difficulties in managing PLWD/carers'/family members' expectations.^37^, ^38^, ^39^

Prescribing decisions also appeared to be influenced by input from carers, but the extent of this involvement was variable. Shared decision‐making is acknowledged to be a crucial aspect of person‐centred dementia care.^40^, ^41^ Increasingly, family members and carers may become more involved with and influence decision‐making, although this is acknowledged to be both difficult and stressful for surrogate decision‐makers.^40^, ^42^, ^43^ These feelings of stress and burden associated with decision‐making were evident in forum users' posts, with many describing they felt pressure to make the right decisions or guilt over making the wrong ones. PLWD and carers' own knowledge and perceptions surrounding the use of (and risks associated with) anticholinergic medications also appeared to influence their ability to contribute to shared decision‐making, and it would be prudent for clinicians to ensure that PLWD and their carers/family members are adequately informed during the decision‐making process and involved from an early stage so that their views and opinions can be fully discussed and considered as part of advance care planning.^12^, ^44^, ^45^

The outcomes from prescribing focused on the effects of the medications and further decision‐making on their prescribing. Forum users described a range of different effects on the PLWD following the use of anticholinergic medications, which varied from positive to negative. These accounts only represent these forum users' perspectives and may not be generalisable to the wider population of PLWD, as the effect of a medication may vary from person to person. While the negative effects of anticholinergic medications are acknowledged within the literature, there may be scenarios in which these medications are appropriately indicated and have a beneficial effect; such experiences were also described by forum users. This study also described many negative effects on carers, which were caused by the presence of noncognitive symptoms and the effect of anticholinergic medications on PLWD. This is consistent with many studies reporting the negative effects of caring on the carers of PLWD.^46^, ^47^ Teahan et al.^48^ reported that family carers of PLWD experienced additional challenges due to the stigma associated with a person receiving a dementia diagnosis and dealing with noncognitive symptoms.^48^ To reduce the carer burden, NICE recommends providing psychoeducation and skills training interventions to carers of PLWD, which includes the provision of advice on how to look after their own physical and mental health, their emotional and spiritual wellbeing and training to help them provide care, such as how to understand and respond to changes in behaviour.^12^ Strengthening the provision of carer support services could reduce the burden and stress among carers of PLWD.^49^

The effects observed with the medication led to further consideration about whether to continue, change or discontinue the medication. Findings from a recent trial reported that implementing medication reviews in routine care could achieve long‐term benefits by increasing the continuity of care for this population.^50^ During the review process, it is crucial to pay attention to the presence of potentially inappropriate medications for PLWD, such as anticholinergic medications.^33^, ^34^, ^51^ When deprescribing of anticholinergic medications took place, this was often reported to be instigated by carers/PLWD reducing the dose or withdrawing the medication themselves. This may happen after experiencing an adverse event if the PLWD or carer is feeling confused or distressed or has concerns about the long‐term effects of a medication or if one believes the issue for which the medication was originally prescribed has been resolved completely.^20^, ^52^, ^53^ However, it would be preferable and good practice for HCPs to oversee the deprescribing, withdrawal or dose reduction of anticholinergic medications in this population so that this can be done in a safe manner.^12^, ^33^, ^34^ There is evidence that reducing the use of anticholinergic medications can reduce carer burden and reduce the frequency, severity and disruptiveness of moderate‐intensity noncognitive symptoms in PLWD.^54^, ^55^

### 4.1 Strengths and limitations

This study utilised a novel method of data collection, and the findings have added to a limited evidence base on carer experiences and perspectives of the use of anticholinergic medications in PLWD. The searches were comprehensive and designed to identify as many relevant posts as possible by using search terms informed by the literature.^8^, ^10^, ^21^ Despite this, it is possible that some potentially relevant posts may not have been identified due to spelling or typographical errors made by forum users, particularly with medication names. Due to limitations with the search facility available on the Talking Point website, advanced search strategies (e.g., truncation, Boolean operators) could not be used, which may have helped to make the search more focused. All posts appeared to be reported by carers, so the perspective of PLWD is absent. And the findings only represent the experiences of those carers who engaged with Talking Point and may not be generalisable to the wider carer population.

## 5 CONCLUSION

This study has provided unique insights into carers' experiences and perspectives about the use of anticholinergic medications in PLWD. The findings have highlighted how commonly these medications are prescribed for PLWD and carers' concerns about their use. There is a clear need for the provision of information about these medications for carers and, indeed, PLWD. Further work is also needed to explore the views and experiences of relevant HCPs so that greater understanding can be sought of how they can contribute to reducing ACB in this population.

## AUTHOR CONTRIBUTIONS

**Bara'a Shawaqfeh**: Conceptualisation; methodology; investigation; formal analysis; writing—original draft; writing—review and editing; visualisation; project administration; funding acquisition. **Carmel Hughes**: Conceptualisation; methodology; formal analysis; writing—original draft; writing—review and editing; visualisation; supervision. **Bernadette McGuinness**: Conceptualisation; writing—review and editing; supervision. **Heather Barry**: Conceptualisation; methodology; formal analysis; writing—review and editing; visualisation; supervision.

## CONFLICT OF INTEREST STATEMENT

The authors declare no conflict of interest.

## ETHICS STATEMENT

Ethical approval was granted for this study by the Faculty of Medicine, Health and Life Sciences Research Ethics Committee, QUB, on 4 January 2022 (Reference MHLS 21_160).

## Supporting information

Supporting information.

## Data Availability

Research data are not shared. Access to the data is not available. Permission to use this data for this study was granted to us from the Talking Point discussion forum (established by the Alzheimer's Society), which is the custodian of the data. All enquiries regarding access to data should be directed to the Alzheimer's Society https://forum.alzheimers.org.uk/.
