# Supplementary material for: Carers' experiences and perspectives of the use of anticholinergic medications in people living with dementia: Analysis of an online discussion forum
Source: Health Expect. 2024 Jan 15;27(1):e13972. doi: 10.1111/hex.13972 (PMC10788817; doi:10.1111/hex.13972)
Supplement: Supplementary file 1 — Supporting information. [file HEX-27-e13972-s001.docx]

#### **Supplementary File 1: Consolidated criteria for reporting qualitative studies (COREQ): 32-item checklist**

| **No** | **Item** | **Guide questions/description** |
| --- | --- | --- |
| **Domain 1: Research team and reflexivity** | | |
| Personal Characteristics | | |
| 1. | Interviewer/facilitator | One researcher (BS) carried out the data extraction |
| 2. | Credentials | BS had BSc and MSc degree in Pharmacy, was a PhD candidate and a qualified pharmacist. Other members of the team had primary degrees in Pharmacy (CMH, HEB) and Medicine (BMcG; specialism in geriatric medicine) and higher research degrees (PhD-CMH, BMcG and HEB; MD- BMcG) |
| 3. | Occupation | BS was a full-time PhD student at the time of  the research study. CMH, BMcG and HEB are academics. |
| 4. | Gender | Male |
| 5. | Experience and training |  |
| Relationship with participants | | |
| 6. | Relationship established | No relationships were established, the researcher acted passively |
| 7. | Participant knowledge of the interviewer | The participants did not have any knowledge about the interviewer as the researcher acted passively |
| 8. | Interviewer characteristics | The researcher had an interest in the research topic of ACB among PwD |
| **Domain 2: Study design** | | |
| Theoretical framework | | |
| 9. | Methodological orientation and Theory | The search terms for data extraction were based on an extensive review of the published literature within the field. Inductive thematic analysis was conducted. |
| Participant selection | | |
| 10. | Sampling | Not applicable |
| 11. | Method of approach | Searching the Talking Point forum using specific search terms |
| 12. | Sample size | Not applicable |
| 13. | Non-participation | Not applicable |
| Setting | | |
| 14. | Setting of data collection | Data was collected via searching the forum using the advanced search facility provided by the forum |
| 15. | Presence of non-participants | Not applicable |
| 16. | Description of sample | Forum users registered in Talking Point forum for People who are affected by dementia or know someone who have dementia |
| Data collection | | |
| 17. | Interview guide | Not applicable |
| 18. | Repeat interviews | Not applicable |
| 19. | Audio/visual recording | Not applicable |
| 20. | Field notes | Not applicable |
| 21. | Duration | Not applicable |
| 22. | Data saturation | Data saturation was reached by the included posts |
| 23. | Transcripts returned | Not applicable |
| **Domain 3: Analysis and findings** | | |
| Data analysis | | |
| 24. | Number of data coders | One researcher (BS) independently coded the data with 20% of the data were coded independently by another researcher (HEB) |
| 25. | Description of the coding tree | Codes represent factors expressed by participants that affected by the use of anticholinergic medications |
| 26. | Derivation of themes | Themes were derived from the data. |
| 27. | Software | Microsoft Word |
| 28. | Participant checking | Not applicable |
| Reporting | | |
| 29. | Quotations presented | Quotations have been presented throughout the Results section of the paper, with identifiers removed.  Each participant was given an anonymous code such as TP001, TP002, TP003 |
| 30. | Data and findings consistent | See the Results section of the paper. We endeavoured to report the study findings in a clear, consistent manner in order to accurately reflect the data that has been collected |
| 31. | Clarity of major themes |  |
| 32. | Clarity of minor themes |  |
